# Measurement resources for dissemination and implementation research in health

**DOI:** 10.1186/s13012-016-0401-y

**Published:** 2016-03-22

**Authors:** Borsika A. Rabin, Cara C. Lewis, Wynne E. Norton, Gila Neta, David Chambers, Jonathan N. Tobin, Ross C. Brownson, Russell E. Glasgow

**Affiliations:** 1 Department of Family Medicine and Public Health, School of Medicine, University of California San Diego, 8452 La Jolla Shores Dr., La Jolla, San Diego, CA 92037 USA; 2 Department of Family Medicine and Adult and Child Center for Health Outcomes Research and Delivery Science, School of Medicine, University of Colorado, Mail Stop F496, Academic Office 1, 12631 East 17th Avenue, Aurora, Denver, CO 80045 USA; 3 Department of Psychological and Brain Sciences, Indiana University, 1101 E. 10th St., Bloomington, IN 47405 USA; 4 Division of Cancer Control and Population Sciences, National Cancer Institute, BG 9609 MSC 9760, 9609 Medical Center Drive, Bethesda, MD 20892-9760 USA; 5 Clinical Directors Network, Inc. (CDN), Center for Clinical and Translational Science, The Rockefeller University, 5 West 37th Street – 10th Floor, New York, NY 10018 USA; 6 Prevention Research Center in St. Louis, Brown School, Washington University in St. Louis, St. Louis, MO 63130 USA; 7 Division of Public Health Sciences and Alvin J. Siteman Cancer Center, Department of Surgery, Washington University School of Medicine, Washington University in St. Louis, St. Louis, MO 63110 USA

**Keywords:** Measurement, Dissemination, Implementation, Resources, Systematic review

## Abstract

**Background:**

A 2-day consensus working meeting, hosted by the United States National Institutes of Health and the Veterans Administration, focused on issues related to dissemination and implementation (D&I) research in measurement and reporting. Meeting participants included 23 researchers, practitioners, and decision makers from the USA and Canada who concluded that the field would greatly benefit from measurement resources to enhance the ease, harmonization, and rigor of D&I evaluation efforts. This paper describes the findings from an environmental scan and literature review of resources for D&I measures.

**Findings:**

We identified a total of 17 resources, including four web-based repositories and 12 static reviews or tools that attempted to synthesize and evaluate existing measures for D&I research. Thirteen resources came from the health discipline, and 11 were populated from database reviews. Ten focused on quantitative measures, and all were generated as a resource for researchers. Fourteen were organized according to an established D&I theory or framework, with the number of constructs and measures ranging from 1 to more than 450. Measure metadata was quite variable with only six providing information on the psychometric properties of measures.

**Conclusions:**

Additional guidance on the development and use of measures are needed. A number of approaches, resources, and critical areas for future work are discussed. Researchers and stakeholders are encouraged to take advantage of a number of funding mechanisms supporting this type of work.

**Electronic supplementary material:**

The online version of this article (doi:10.1186/s13012-016-0401-y) contains supplementary material, which is available to authorized users.

## Background

Measurement issues often threaten the evolution of new fields [1]. Recent reviews suggest that fewer than 50 % of existing dissemination and implementation (D&I) research measures are psychometrically validated (i.e., in many cases, no data exists on whether the measure assesses the construct it is intended to address; [2–4]). In addition to psychometric quality, information about pragmatic quality, including clinical or operational utility, is gaining ground as an important measurement dimension [5], particularly for advancing the practice of D&I [6]. Additional challenges include the dearth of measures available for certain D&I constructs (e.g., context, adaptation) [7]. However, perhaps the most critical challenge is the combination of these issues: the apparent lack of pragmatic and high-quality measures for key constructs and the disparate use of measures across studies, which inhibits the integration of results from observational and interventional studies conducted across multiple sectors that examine health behaviors and outcomes.

D&I research is especially vulnerable to communication barriers that may exacerbate these measurement issues given the rapid spread of the field across numerous disciplines within and outside of healthcare (e.g., healthcare, mental health, public health, education). A likely issue is that D&I scientists working in clinical medicine or healthcare may not be aware of measures available from those working in public health or mental health, for example. Without shared measures resources, the field is vulnerable to redundancies in measure development and missed opportunities to use common measures across studies, with the ultimate consequence being an artificially fractured knowledge base and inefficient efforts to advance the field.

From 2007 to 2012, the USA (US) National Institutes of Health (NIH) held five large conferences on D&I research, choosing themes that would call attention to aspects of the field for which advances were particularly needed. While the large meetings, including one specifically focusing on research methods and measurement, were able to spotlight the “state-of-the-science” across different domains to guide development, there was limited opportunity to directly fill the gaps that speakers had identified. In 2013, the NIH developed a series of three meetings with the purpose to convene working groups of leaders in D&I research to identify gaps, articulate key next steps, and locate potential tools for the field related to (1) training, (2) study design, and (3) measurement and standardized reporting. On October 23–24, 2013, the working group on measurement and standardized reporting, including 23 representatives from large-scale efforts to synthesize and evaluate D&I measures—including from the Society for Implementation Research Collaboration (SIRC), the US National Cancer Institute (NCI) Grid-Enabled Measures D&I campaign, and the affiliated NIH Clinical and Translational Science Award Community-Engaged Research and Comparative Effectiveness Research measurement effort—took on the challenge of assessing the state of D&I measures and of identifying mechanisms to improve standardized reporting across studies. In bringing together this group of scientists, it became quickly apparent that cross-talk between different research areas was lacking, not only with respect to the use of similar measures but also in terms of knowledge of measure resources that could promote ease of measure identification, selection, and harmonization. Thus, a subgroup of scientists from this meeting aimed to locate existing measure resources to share with D&I-engaged scientists and to reveal action steps that emerged from the meeting with a focus on measure development grants as a potential avenue for filling the obvious gaps in the field.

## Findings

### Method

#### Review objective and scope

The primary goal of this paper is to provide a review of existing measure resources relevant to D&I research and to describe their characteristics and possible use by researchers and other practitioners/end-users. Specifically, the target was resources that provided information about D&I measures (i.e., websites or systematic reviews that synthesized information about existing D&I measures), not individual D&I measures as these are captured within the resources we sought to identify. Measure resources include living repositories (e.g., websites and wiki pages) and static resources (e.g., systematic and scoping reviews).

#### Resource identification

To increase the comprehensiveness and exhaustiveness of the search, we employed a two-step search process that concluded in May 2015. First, we conducted an environmental scan using a respondent-driven, non-probabilistic sampling approach to identify key informants who could help us identify additional resources beyond the peer-reviewed literature, in the gray literature or in the development stages. This approach, which leverages the informational power of social networks, can augment traditional environmental scans and literature reviews in situations in which the searched-for items (i.e., measures resources) are not clearly and consistently indexed with standard terms in bibliographic databases. The following listservs were accessed: the SIRC, the Association for Behavioral and Cognitive Therapy Dissemination and Implementation Science Special Interest Group, and the Implementation Network listserv. We also searched websites and electronic newsletters for additional resources.

Second, a review (scoping and systematic) of the published literature was conducted using two approaches. First, an initial set of publications (reviews of dissemination and/or implementation measures) was identified through recommendations from attendees of the NIH meeting. Then, a systematic review of the literature was completed using two search engines (PubMed, Web of science) to identify papers published between 2000 and 2014 in English language using a set of search term combinations (dissemination/implementation + measure/measurement/instrument/scale/evaluation + review). Titles and abstracts were filtered for reviews (both systematic and non-systematic) of dissemination and/or implementation measures.

#### Resource inclusion/exclusion

Five inclusion/exclusion criteria were set. First, the resources needed to include measures related explicitly to D&I. Therefore, resources that included information about measures used to evaluate D&I outcomes were included, whereas resources focused solely on quality improvement or quality of care measures or patient-level health outcomes were excluded. Second, resources were excluded if the investigative team was unable to access the resource beyond its cited name or if the resource was not yet fully developed. Third, for static resources, we included published reviews (systematic or not) that focused on one or multiple D&I-relevant constructs, which includes D&I outcomes (e.g., adoption, sustainment; [8]) or factors implicated in the D&I process (e.g., leadership, climate; [9]). Fourth, only reviews were included from the published literature. Finally, resources that discussed either/both quantitative and qualitative measures were included.

#### Data extraction

The focus of the data extraction was collaboratively developed to obtain useful summary information that could be gleaned from the resources and would be applicable across resources, to ultimately aid researchers and stakeholders in determining the resource of most relevance. The data extraction resulted in 13 unique pieces of data that reflected both quantitative and qualitative information about the resources including characterizing features of the resource (organizing framework, audience, discipline/scope, type of measure, measure identification approach, resource status, and access) and summary data of the measures information (number of constructs, number of measures, if the measures are included in the resource, measure metadata, psychometric information, pragmatic rating, and analysis level).

Once the resource sample and the data extraction process were finalized, the data extraction was completed by two independent research assistants trained by the first authors (BR and CCL). Research assistants independently extracted data from each resource and then met for consensus in order to achieve one set of summary data for each resource [10]. When consensus could not be achieved, the first authors were consulted to make a final determination.

## Results

A total of 17 measure resources were included in the review and subjected to data extraction to obtain summary information that may aid end-users in identifying and selecting quantitative and qualitative measures related to D&I (see Tables 1 and 2). Twelve resources were static reviews and five were web-based resources, the latter of which are reported to be “living” in that they continue to be updated with the literature base. Fourteen of the 17 resources are publicly available, requiring no membership or application process to view or use. For seven of these resources, this also means that the measures themselves are publicly available. The majority of the identified resources are accessible at no cost, except for the two reviews that are not published in open-access journals and one web-based resource that requires paid membership but for which the results are also available in an open source peer-reviewed publication [2].

**Table 1:** Characterizing features of measures resources

Resource	Resource status	Access	Discipline/scope	Measure identification approach	Type of measure(s)	Target audience	Organizing framework
Ready, set change! Online decision support tool http://www.ncbi.nlm.nih.gov/pubmed/24886072	Living	Open	Health	Database review, Experts contacted	Quan	Implementers, Researchers	Readiness for change [15]
Proxy measures of clinical behavior http://www.ncbi.nlm.nih.gov/pubmed/19575790	Static	Open	Health	Database review	Quan	Health policy makers, Practitioners, Researchers	NS
Organizational readiness for change measures http://mcr.sagepub.com/content/65/4/379.abstract	Static	Limited	Business, Education, Health, Human Service/Government	Database review	Quan + Qual	Implementers, Researchers	Organizational readiness for change [16]
Clinical community relationships measures database http://primarycaremeasures.ahrq.gov/clinical-community/	Static	Open	Health	Crowd sourced, Database review, Experts contacted, Snowball sampling	Quan + Qual	Practitioners, Researchers	Clinical community relationships measures framework [17]
Eurocontrol change and transition tools https://www.eurocontrol.int/sites/default/files/content/documents/nm/safety/safety-change-and-transition-tools-compendium-main-document-2010.pdf	Static	Open	Business	Experts contacted	Quan	Implementers, Researchers	Proactive aviation change and transition process model [18]
Team-based primary care measures database http://primarycaremeasures.ahrq.gov/team-based-care/	Static	Open	Health	Crowd sourced, Database review, Experts contacted, Snowball sampling	Quan	Practitioners, Researchers	The conceptual framework of team-based primary care [19]
Systems antecedents for dissemination and implementation: a review and analysis of measures http://www.ncbi.nlm.nih.gov/pubmed/21724933	Static	Open	Health	Database review	Quan	Implementers, Researchers	Conceptual model for considering the determinants of diffusion, dissemination, and implementation of innovations in health service delivery and organization [5]
Measuring factors affecting implementation of health innovations http://www.ncbi.nlm.nih.gov/pubmed/23414420	Static	Open	Health	Database review	Quan	Implementers, Researchers	Five-factor framework: 1) Structural; 2) Organizational; 3) Patient; 4) Provider; 5) Innovation-level
Measures for predictors of innovation adoption http://www.ncbi.nlm.nih.gov/pubmed/24740175	Static	Open	Mental health	Snowball sampling	Quan + Qual	Implementers	CFIR [9]
Organizational context measures http://www.ncbi.nlm.nih.gov/pubmed/19454008	Static	Open	Health	Database review	Quan	Practitioners, Researchers	Framework of four domains: 1) Research utilization; 2) Research activity; 3) Knowledge management; 4) Organizational learning
Theoretical domains framework for SCP use http://www.ncbi.nlm.nih.gov/pmc/articles/PMC4236456/	Static	Limited	Health	Experts contacted	Quan + Qual	Implementers, Researchers	Theoretical domains framework [20]
Knowledge translation: introduction to models, strategies, and measures http://ktdrr.org/ktlibrary/articles_pubs/ktmodels/index.html	Static	Open	Education, Health	Database review	Quan	Health policy makers, Practitioners, Researchers	CIHR model of knowledge translation [21]
Fidelity measures for mental health http://www.ncbi.nlm.nih.gov/pubmed/22854723	Static	Open	Mental health	NS	Quan + Qual	Practitioners, Researchers	NS
GEM D&I https://www.gem-measures.org/public/wsoverview.aspx?wid=11&cat=8&aid=0	Living	Open	Health	Crowd sourced	Quan	Implementers, Practitioners, Researchers	NS
Registry of knowledge translation methods and tools http://www.nccmt.ca/registry/index-eng.html	Living	Open	Health	Database review, Experts contacted	Quan + Qual	Practitioners	CIHR model of knowledge translation [21]
SIRC instrument review project http://www.societyforimplementationresearchcollaboration.org/sirc-projects/sirc-instrument-project/	Living	Limited	Mental health	Database review, Snowball sampling	Quan	Implementers, Researchers	CFIR [9] and implementation outcomes [8]
CFIR technical assistance website http://cfirguide.org/	Living	Open	Health	Crowd sourced, Experts populated	Quan + Qual	Implementers, Researchers	CFIR [9]

*Note: Resource* provides the name of the resource and a hyperlink to either the resource website or the PubMed abstract for the article. *Resource status* refers to whether the resource is living or static, where “living” represents a resource that evolves with the literature and “static” represents a resource that reflects the status of measurement at the point of publication. *Access* describes the accessibility of the resource, such as whether special permission is required for viewing the resource. For this code, “Open” refers to resources that are publicly available, while “Limited” signals that a journal subscription or membership is required to access the resource. *Discipline/scope* displays the field(s) from which the resource was generated. *Measure identification approach* provides the way in which the resource described its approach for identifying its included measures. *Type of measure(s)* describes the type of measures that are included in the resource. For this code, Quan = quantitative, Qual = qualitative, Quan + Qual = both quantitative and qualitative. *Target audience* describes the audience who the resource is aiming to assist. *Implementers* refers to stakeholders leading the implementation. *Organizing framework* provides the framework the resource listed as that which informed their identification and organization of the measures they included and its associated citations from which the constructs come from (see corresponding numbered reference for citation)

**Table 2:** Summary data of measures information

Resource	# of constructs	# of measures	Measures included	Measure metadata	Psychometric information	Pragmatic rating	Analysis level
Ready, set change! Online decision support tool http://www.ncbi.nlm.nih.gov/pubmed/24886072	4	9	Yes	Author/date, instrument citation, instrument link, instrument setting, total number of items	NS	Yes	Organization
Proxy measures of clinical behavior http://www.ncbi.nlm.nih.gov/pubmed/19575790	1	15	No	Author/date, instrument citation	Reliability: inter-rater; test-retest Validity: external	No	Consumer, organization, Provider, System, Team
Organizational readiness for change measures http://mcr.sagepub.com/content/65/4/379.abstract	1	43	No	Author/date, instrument citation, instrument setting	Reliability: inter-item; inter-rater; parallel forms; test-retest Validity: concurrent; convergent; discriminate; face/content; predictive	No	Organization
Clinical community relationships measures Database http://primarycaremeasures.ahrq.gov/clinical-community/	9	22	Yes	Author/date, instrument citation, instrument link, instrument setting, purpose/definition	NS	No	Consumer, organization, Provider
Eurocontrol change and transition tools https://www.eurocontrol.int/sites/default/files/content/documents/nm/safety/safety-change-and-transition-tools-compendium-main-document-2010.pdf	NS	29	Yes	Author/date, cost, instrument citation, language, purpose/definition, total number of items	Reliability: internal consistency; test-retest Validity: construct; content; face	Yes	Consumer, organization, Provider, System, Team
Team-based primary care measures database http://primarycaremeasures.ahrq.gov/team-based-care/	12	48	Yes	Author/date, instrument citation, instrument link, instrument setting, link to articles citing instrument, mediator constructs, purpose/definition, total number of items	NS	No	Team
Systems antecedents for dissemination and implementation: a review and analysis of measures http://www.ncbi.nlm.nih.gov/pubmed/21724933	5	36	No	Author/date, instrument setting, link to articles citing instrument, total number of items	Reliability: internal consistency; inter-rater; parallel forms; test-retest Validity: predictive; face/content; concurrent; convergent; discriminate	No	Organization, Provider
Measuring factors affecting implementation of health innovations http://www.ncbi.nlm.nih.gov/pubmed/23414420	5	62	Yes	Instrument citation, instrument setting	NS	No	Consumer, organization, Provider, System, Team
Measures for predictors of innovation adoption http://www.ncbi.nlm.nih.gov/pubmed/24740175	27	118	No	Instrument citation, purpose/definition	NS	No	Consumer, organization Provider, System, Team
Organizational context measures http://www.ncbi.nlm.nih.gov/pubmed/19454008	18	18	No	Instrument citation	NS	No	Organization, Provider, System, Team
Theoretical domains framework for SCP use http://www.ncbi.nlm.nih.gov/pmc/articles/PMC4236456/	112	1	Yes	NS	NS	No	Consumer, organization Provider, System, Team
Knowledge translation: introduction to models, strategies, and measures http://ktdrr.org/ktlibrary/articles_pubs/ktmodels/index.html	1	6	No	Author/date, links to articles citing instrument	NS	No	Consumer, organization Provider, System, Team
Fidelity measures for mental health http://www.ncbi.nlm.nih.gov/pubmed/22854723	1	4	No	Author/date, instrument citation, instrument setting, purpose/definition	NS	No	Organization, provider, Team
GEM D&I https://www.gem-measures.org/public/wsoverview.aspx?wid=11&cat=8&aid=0	359	895	Yes	Author/date, instrument citation, language, purpose/definition, total number of items	Reliability: internal consistency Validity	Yes	Consumer, organization, Provider, System, Team
Registry of knowledge translation methods and tools http://www.nccmt.ca/registry/index-eng.html	NS	191	Yes	Author/date, cost, instrument citation, language, PubMed abstract, purpose/definition, total number of items	NS	No	Consumer, organization, Provider, System, Team
SIRC instrument review project http://www.societyforimplementationresearchcollaboration.org/sirc-projects/sirc-instrument-project/	33	>450	Yes	Instrument citation, link to articles citing instrument, purpose and/or definition, sample items	Norms Reliability: internal consistency Validity: structural; predictive Responsiveness	No	Consumer, organization, Provider, System, Team
CFIR technical assistance website http://cfirguide.org/	33	6	Yes	Author/date, mediator constructs, PubMed abstract, total number of items	NS	No	Organization, provider, System, Team

*Note: Resource* provides the name of the resource and a hyperlink to either the resource website or the PubMed abstract for the article. *# of constructs* provides the number of constructs used to organize the resource. *# of measures* provides the number of measures that are included in the resource. *Measures included* indicates whether the resource includes actual measures or simply provides information about them. *Measure metadata* describes the information that is provided about the measures that are included in the resource. *Psychometric information* describes the psychometric property information that is provided for each measure within the resource. *Pragmatic rating* indicates whether the resource included a rating of pragmatic qualities for the included measures. *Analysis level* indicates with whom the included measures were intended to be used

Three resources came from mental health, one from business, and the remaining from health disciplines. Eleven reported identifying measures from databases, of which five combined this search strategy with a second approach (e.g., expert review, snowball sampling); the remaining drew from experts, snowball sampling, crowd sourcing, or did not specify their search approach (*n =* 6). Ten provided information on quantitative measures only, seven on both quantitative plus qualitative. All resources were developed for use by researchers with implementation practitioners as the next most commented target audience (*n =* 9).

Fourteen resources were organized according to an established D&I theory or framework. Most notably, the Consolidated Framework for Implementation Research (CFIR) [9] was represented in three of the resources. The number of constructs ranged from 1 to 359. A similar broad range was observed in the number of measures: 1 to >450. Although only nine resources provided the measure, 11 resources provided information regarding measure citations to promote ease of access. The amount and type of information provided regarding both the measures themselves (i.e., metadata) and the development and validation of the measures was also quite variable. For example, six out of the 17 resources included reliability and validity information and only three resources provided information about pragmatic measure qualities. Ten provided measures targeting consumers and 14 targeting providers.

## Discussion

In this short report, we described 17 resources that synthesized and evaluated existing measures for D&I research. These resources included four web-based repositories and 13 static reviews or tools, each providing varying levels of measure metadata. The summary of the resources provided herein can be used as a starting point for researchers and other stakeholders (e.g., implementation practitioners, administrators) intending to identify measures for D&I studies. In the case of interactive resources in which crowd sourcing of data and experiences is encouraged, end-users can share experiences and additional measure metadata. Taken together, these can facilitate a culture of measure harmonization and data comparison across studies [11]. For example, the SIRC Instrument Review Project provides expert-informed rating of measures organized along the CFIR [9] and the Implementation Outcomes Framework [8] making the identification of scientifically sound D&I measurement convenient [2]. Another interactive instrument, the D&I Workspace in the Grid-enabled Measures Database uses a crowd sourcing approach to populate, update, rate, and comment on measures that are organized around critical D&I constructs [12]. Active participation from researchers and practitioners in the development and refinement of these and other interactive resources for D&I measures is critical for achieving their ultimate goal of being living and relevant resources for the D&I community.

One important shortcoming of these resources and the D&I field in general was identified through a conceptual framework that emerged as one of the main products of the NIH D&I measurement and reporting standards working meeting. This framework revealed a number of D&I-relevant constructs (e.g., context, sustainability, evolution) which lack appropriate measures as well as constructs for which measures exist but are not commonly used (e.g., cost of intervention, adoption, implementation strategy) [7]. For these areas, additional measure development and guidance on the development and use of measures are needed. A number of approaches and resources are in place to support additional measure development.

1. Generation of single-use measures (i.e., developed “in-house” for use in a specific setting or context) remains the status quo [1]; however, informed by the working meeting, an effort is underway to generate pragmatic measures with strong psychometrical properties of three implementation outcomes that predict adoption for use across studies as well as a replicable measure development process [6].
2. A web-based interactive tool (another product emerging from this NIH D&I meeting) provides guidance for the selection, adaptation, and integration of D&I models and also allows for the linkage of model constructs to existing measures [13].
3. A working group, Qualitative Research in Implementation Science (QUALRIS) was assembled by the NCI’s Implementation Science team including national experts in qualitative and mixed methods research to develop guidelines and standards for the use of qualitative methods for D&I research (S. Heurtin-Roberts, personal communication, September 28, 2015).
4. Funding mechanisms for the advancement of D&I measurement are in place. There are three main venues through which researchers in the USA have been supported for measure development (examples for each venue are provided in Additional file 1):
  I. Research funding announcements have included an explicit focus on measure development as the major activity within a grant or contract. For example, the standing NIH D&I program announcements have consistently called for “Development of D&I-relevant outcome and process measures and suitable methodologies for dissemination and implementation approaches that accurately assess the success of an approach to move evidence into practice” (NIH, PAR-13-055, PAR-13-054, PAR-13-056). Grant applications could thus propose to develop and test a novel D&I instrument as the central aim of the study. Similarly, PCORI’s Program Funding Announcements on Communication and Dissemination Research and Improving Methods for conducting Patient-Centered Outcomes Research includes solicitation of “Studies to develop and compare alternative methods and tools to elicit and include patient-desired outcomes in the healthcare decision-making process” (PCORI, 2013) and “projects to address gaps in methodological research relevant to conducting patient-centered outcomes research (PCOR).” (http://www.pcori.org/funding-opportunities/announcement/improving-methods-conducting-patient-centeredoutcomes-research-3).
  II. Multiple funders have enabled researchers to include measurement development for key outcomes of a prospective trial to be included as part of study development.
  III. Finally, funding announcements have developed opportunities for measure development as part of a broader set of activities. The US Veterans Affairs’ Health Services Research and Development program, for example, review implementation science relevant applications and allow measurement development to exist within these applications. Furthermore, the Quality Enhancement Research Initiative (QUERI) mechanisms allow for the addition of research protocols on to an existing quality improvement project that is part of the QUERI program or a partnered evaluation as long as it fits the main program’s impact goal and the needs of the operational partner [14]. The NIH Institutes and Centers have frequently included methods and measurement cores as components of research centers, conference grants (SIRC, for example, began through an NIMH-funded conference grant, 5R13MH086159-05, https://projectreporter.nih.gov/project_info_description.cfm?aid=8645741&icde=26765462&ddparam=&ddvalue=&ddsub=&cr=2&csb=default&cs=ASC) and other infrastructure mechanisms.

## Conclusion

Advancing and strengthening measurement approaches for D&I research are critical to building a cumulative scientific knowledge base and offering tools for informing the real-world practice of D&I. A number of existing measurement resources can provide a starting point to researchers and stakeholders for the identification of appropriate measures and harmonization of measurement use across studies. However, additional work needs to take place to advance and strengthen the field. Critical areas for development include the following: additional high quality, pragmatic measures for key D&I-related constructs for which measures do not exist; a core set of brief measures that can be used efficiently across pragmatic clinical trials and practice-based observational studies; and a rapid-cycle measure development process. Researchers and stakeholders are encouraged to take advantage of a number of funding mechanisms supporting this type of work. Implementation of a set of core measures across multiple studies would facilitate future synthesis enabling the examination of the impact of various D&I constructs on clinical and population health outcomes.

## Additional file

Additional file 1: Examples of funding mechanisms for the advancement of D&I measurement. (DOCX 21 kb)

## Abbreviations

CFIR: Consolidated Framework for Implementation Research
D&I: Dissemination & Implementation
EBP: evidence-based practice
NCI: National Cancer Institute
NIH: National Institutes of Health
NIMH: National Institutes of Mental Health
PCOR: Patient-Centered Outcomes Research
PCORI: Patient-Centered Outcomes Research Institute
QOL: quality of life
QUALRIS: Qualitative Research in Implementation Science
QUERI: Quality Enhancement Research Initiative
SIRC: Society for Implementation Research Collaboration
