# Supplementary material for: Measurement resources for dissemination and implementation research in health
Source: Implement Sci. 2016 Mar 22;11:42. doi: 10.1186/s13012-016-0401-y (PMC4802882; doi:10.1186/s13012-016-0401-y)
Supplement: Additional file 1: — Examples of funding mechanisms for the advancement of D&I measurement. (DOCX 21 kb) [file 13012_2016_401_MOESM1_ESM.docx]

Additional file 1: Examples of funding mechanisms for the advancement of D&I measurement

**Examples of funding mechanisms for the advancement of D&I measurement**

Here we provide three examples, one for each type of funding mechanism described in the short report, of funded research in D&I measure development.

Type 1: Research funding announcements with **explicit focus on measure development as the major activity within a grant or contract**.

This example is an R21 developmental research grant, in response to the Trans-NIH Dissemination and Implementation Research in Health Program Announcement (PAR-13-055, http://grants.nih.gov/grants/guide/pa-files/PAR-13-055.html) The investigators proposed to develop contextual measures for organizational settings, focusing on strategic climate, that would assess the degree to which organizational context is a barrier or facilitator to effective implementation of an evidence-based program. Specifically, they proposed to develop a set of quantitative measures that can be used to assess implementation climate, implementation leadership, and implementation citizenship behavior. The development of such measures included assessing the factor structure, reliability, and construct and criterion-related validity of the measures, and then assessing generalizability by validating the factor structure across multiple public sector service settings, in this case: mental health, child welfare, and alcohol/drug services. The development of these quantitative measures has the potential to advance implementation science by providing metrics to better understand the role of organizational context and to assist decision makers to more effectively evaluate organizational preparedness for evidence-based program implementation [1].

Type 2: I**nclusion of measurement development for key outcomes of a prospective trial as part of study development**

This example is that of measure development within the context of an R01 research study (R01MH076158-01A1); in this example, the measure provided a necessary tool to conduct the research study. The measure that was developed is an observational measure of implementation progress, called the Stages of Implementation Completion (SIC) [2]. The SIC measure assesses attainment time of key implementation milestones and the proportion of activities completed in the implementation of an EBP. The measure was developed in a randomized controlled trial comparing the effectiveness of two implementation strategies, and tracked the progress of eight implementation stages, each of which was defined by a set of specific activities [2]. The investigators are further evaluating SIC for construct and external validity as well as reliability in a subsequent research grant [3].

Type 3: Funding announcements for **measure development as part of a broader set of activities**

This type of funding for measures development is part of a broader set of activities. One example is the development of a set of quality measures for partnership processes and community engagement as part of the CTSA Consortium (supported by the National Center for Research Resources and the National Center for Advancing Translational Sciences) and the Native American Research Centers for Health (NARCH) Program (supported by the National Institutes of General Medical Sciences [NIGMS] at the NIH) [4] [5] [6] [7]. Through this initiative, investigators developed a set of 22 reliable and valid measures related to the Community-Based Participatory Research (CBPR) conceptual model. These measures were developed through these larger programs with access to data and input from hundreds of federally-funded CBPR projects. These measures, in turn, provide resources not only for CBPR practitioners and researchers, but also for researchers who wish to measure partnership processes and levels of community engagement.

Another example is the support of infrastructure development for the systematic use of person-centered health outcomes measurement systems in health research (RFA-CA-13-008, <http://grants.nih.gov/grants/guide/rfa-files/RFA-CA-13-008.html>). These systems include the Patient Reported Outcomes Measurement Information System® (PROMIS®: <http://www.nihpromis.org/>); the NIH Toolbox for Assessment of Neurological and Behavioral Function (NIH Toolbox: <http://www.nihtoolbox.org/>); the Quality of Life (QOL); Outcomes in Neurological Disorders (Neuro-QOL: <http://www.neuroqol.org/>); and the Adult Sickle Cell Quality of Life Measurement Information System (ASCQ-Me: <http://www.air.org/files/4_pager_AIR_Health_Polict_2011_V10F.pdf>). The resulting Person-Centered Assessment Resource (<http://healthcaredelivery.cancer.gov/pcar/>) offers an integrated platform to incorporate health outcomes within research studies.

**References:**

1. Aarons GA, Ehrhart MG, Farahnak LR: **The implementation leadership scale (ILS): development of a brief measure of unit level implementation leadership**. *Implement Sci* 2014, **9**:45.

2. Chamberlain P, Brown CH, Saldana L: **Observational measure of implementation progress in community based settings: the stages of implementation completion (SIC)**. *Implement Sci* 2011, **6**:1–8.

3. Saldana L: **The stages of implementation completion for evidence-based practice: protocol for a mixed methods study**. *Implement Sci* 2014, **9**:43.

4. Pearson CR, Duran B, Oetzel J, Margarati M, Villegas M, Lucero J, Wallerstein N: **Research for Improved Health: Variability and Impact of Structural Characteristics in Federally Funded Community Engaged Research**. *Prog Community Health Partnersh Res Educ Action* 2015, **9**:17–29.

5. Hicks S, Duran B, Wallerstein N, Avila M, Belone L, Lucero J, Magarati M, Mainer E, Martin D, Muhammad M, others: **Evaluating community-based participatory research to improve community-partnered science and community health**. *Prog Community Health Partnersh Res Educ Action* 2012, **6**:289.

6. Oetzel JG, Zhou C, Duran B, Pearson C, Magarati M, Lucero J, Wallerstein N, Villegas M: **Establishing the psychometric properties of constructs in a community-based participatory research conceptual model**. *Am J Health Promot* 2015, **29**:e188–e202.

7. Eder MM, Carter-Edwards L, Hurd TC, Rumala BB, Wallerstein N: **A logic model for community engagement within the Clinical and Translational Science Awards consortium: can we measure what we model?** *Acad Med J Assoc Am Med Coll* 2013, **88**:1430–1436.
